# Male Gender Is Associated with Lymph Node Metastasis but Not with Recurrence in Papillary Thyroid Carcinoma

**DOI:** 10.1155/2022/3534783

**Published:** 2022-02-28

**Authors:** Jiang Zhu, Rui Huang, Ping Yu, Haoyu Ren, Xinliang Su

**Affiliations:** ^1^Department of Endocrine and Breast Surgery, The First Affiliated Hospital of Chongqing Medical University, Chongqing, China; ^2^Department of Liver Surgery, Liver Transplantation Division, West China Hospital, Sichuan University, Chengdu, China; ^3^Department of Anesthesiology, The Second Affiliated Hospital, Zhejiang University School of Medicine, Hangzhou, China

## Abstract

**Background:**

The incidence of papillary thyroid carcinoma (PTC) is higher in females than in males, but it remains unclear whether gender is associated with the aggressiveness of this disease. We aimed to clarify the influence of gender on the risk of developing lymph node metastasis (LNM) and on the prognosis of PTC patients. *Study Design.* Retrospective cohort study. *Setting*. Academic tertiary care center.

**Methods:**

Clinical data of PTC patients who were admitted to the Department of Endocrine and Breast Surgery, the First Affiliated Hospital of Chongqing Medical University, between January 2013 and December 2018 were retrospectively reviewed. The differences in clinical features and outcomes between female and male patients were compared. Univariate and multivariate logistic regression analyses were conducted to assess the impact of gender on LNM. Kaplan–Meier curves were used to estimate recurrence-free survival (RFS).

**Results:**

A total of consecutive 2536 patients were enrolled in this study. Males accounted for 25.2% (639 cases) of all patients. Central lymph node metastasis (CLNM) and lateral lymph node metastasis (LLNM) rates were 52.5% (1346/2536) and 22.0% (558/2536), respectively. Male presented with higher LNM rates than female patients (65.7% vs. 51.2%; *P* < 0.001). Male gender was independently associated with LNM (OR = 1.93, 95% CI: 1.59–2.35; *P* < 0.001). After full adjustment, male gender still remained significantly associated with CLNM in all subgroups; however, subgroup analyses indicated no significant relationship between gender and LLNM. In addition, after a median follow-up period of 30 months, no significant difference was found in RFS between female and male patients (*P*=0.15).

**Conclusions:**

This observational cohort study revealed that male gender was significantly associated with CLNM; whereas, LLNM was not different between female and male PTC patients in southwestern China. Moreover, currently, there is insufficient evidence to justify that male gender is an independent prognostic factor for recurrence.

## 1. Introduction

The incidence of papillary thyroid carcinoma (PTC) has increased rapidly in recent years [1–3]. Similar to some other tumors [4], a differing incidence of thyroid cancer between genders is apparent. It is well-known that there are approximately three times as many females suffering from thyroid cancer as males [5–7]. However, male gender is reported to be a poor prognostic factor in PTC, with notably higher death and recurrence rates [8–12]. On the contrary, some prior studies suggested that male gender is not associated with tumor aggressiveness or lymph node status [13–15]. Therefore, it is not easy to make an appropriate therapeutic decision according to these contradictory results.

In fact, PTC is a heterogeneous disease [7, 16], and the risk of lymph node metastasis (LNM) varies with the differing clinicopathological features of each patient [17–19]. LNM in PTC patients is common, which may lead to tumor relapse and repeat surgery [20, 21]. To date, the relationship of gender with clinicopathological characteristics and prognosis in PTC patients has not been well defined, particularly regarding LNM, and reports on this issue are limited.

Thus, we aimed to evaluate the impact of gender on LNM and assess the risk of recurrence in subgroups of PTC with various clinicopathological characteristics based on real-world data of thyroid cancer cases from southwest China.

## 2. Methods

### 2.1. Patients

Medical records of patients between January 2013 and December 2018 at the Department of Endocrine and Breast Surgery, the First Affiliated Hospital of Chongqing Medical University, were reviewed and collected. The inclusion criteria were as follows: (a) age 18–80 y, (b) initial thyroid surgery, and (c) histologically diagnosed with PTC. Patients with incomplete clinical data or distant metastasis were excluded from this study.

### 2.2. Treatment and Follow-Up

As described previously, our PTC patients underwent standard treatment procedures: lobectomy plus ipsilateral central compartment lymph node dissection which is recommended by Chinese guideline [22–24]. In our center, intraoperative frozen section of ipsilateral central lymph nodes was routinely used. Total thyroidectomy plus ipsilateral central compartment lymph node dissection was indicated for PTC patients with LNM, extrathyroid extension (ETE), bilaterality, or tumor size >20 mm. The decision to perform lateral lymph node dissection was comprehensive, determined based on preoperative ultrasound-reported LN status, tumor size and location, ETE, and intraoperative frozen section results. Radioactive iodine therapy and thyroid stimulating hormone suppression were recommended for individuals when clinically indicated.

Our preoperative ultrasound report consisted of the following clinicopathological parameters: the location and size of tumor, multifocality, bilaterality, chronic lymphocytic thyroiditis (CLT), and clinical N (cN) stage: cN0, cN1a, and cN1b. The details of intraoperative findings and surgery procedures were carefully recorded by surgeons. All surgical specimens were sent for postoperative histopathology. Pathology reports included tumor size, multifocality, ETE, chronic lymphocytic thyroiditis (CLT), number of central lymph node metastasis (CLNM), and lateral lymph node metastasis (LLNM).

Postoperative patients regularly came back to the outpatient for a check every 3 months for 1 year after initial therapy, followed by one visit every 6 months. The follow-up examination items included serum levels of triiodothyronine (T3), thyroxine (T4), thyrotrophin (TSH), thyroglobulin (Tg), thyroid globulin antibody (TgAb), and neck ultrasound. The definition of recurrent disease was a nonstimulated Tg level of ≥0.2 ng/mL, stimulated Tg > 1 ng/mL, or evidence of disease by neck imaging during follow-up time [12]. Recurrence-free survival (RFS) was defined as the time from the initial surgery to the discovery of cancer recurrence or the most recent follow-up.

### 2.3. Statistical Analysis

Categorical and continuous variables between male and female patients were analyzed using Fisher's exact test and Student's *t*-test, respectively. Multivariable logistic regression analyses with a backward stepdown selection were performed to identify the independent risk factors of lymph node metastasis. We also conducted stratified analyses to assess the relationship between gender and CLNM or LLNM. Kaplan–Meier curves and the log-rank test were used to calculate RFS. Data analyses were performed using *R* software (v. 3.4.3). All statistical tests were considered significant for *P* < 0.05.

## 3. Results

### 3.1. Patient Clinical Pathological Characteristics

Overall, a total of 2536 cases were included in this study. The average age of the total study population was 42.9 ± 11.9 y (mean ± SD). Additionally, 639 (25.2%) were males and 1897 (74.8%) were females. Demographic and clinicopathologic characteristics of patients with PTC are given in Table 1. Papillary thyroid microcarcinoma (PTMC) patients accounted for 56.3% of all patients. ETE and CLT were detected in 865 (34.1%) and 526 (20.7%) patients, respectively. LNM occurred in 54.9% of patients. CLNM and LLNM rates were 52.5% (1346/2536) and 22.0% (558/2536), respectively. Recurrence occurred in 29 patients (1.1%).

The female group had higher percentages of CLT (10.6% vs. 24.1%; *P* < 0.001) than the male group, while CLNM (64.2% vs. 48.6%; *P* < 0.001) and LLNM (26.3% vs. 20.6%; *P* < 0.001) occurred more frequently in the male group. No differences were found in age, tumor size, ETE, multifocality, and recurrence between male and female groups (all *P* > 0.05).

### 3.2. Univariate and Multivariate Logistic Regression Analyses of PTC Patients

Associations between LNM and the potential risk factors are given in Table 2. Univariate analysis showed that male gender, age, tumor size, ETE, multifocality, and bilaterality were significantly associated with LNM. However, tumor location and CLT showed no significant association with LNM. In multivariate logistic regression analysis, male gender (OR = 1.93, 95% CI: 1.59–2.35; *P* < 0.001), larger tumor size (10–20 mm, OR = 2.09, 95% CI: 1.75–2.52; *P* < 0.001) (>20 mm, OR = 4.18, 95% CI: 3.06–5.73; *P* < 0.001), ETE(+) (OR = 1.31, 95% CI: 1.10–1.57; *P*=0.003), and multifocality(+) (OR = 1.92, 95% CI: 1.53–2.41; *P* < 0.001) were independent risk factors of LNM. On the contrary, when compared to age <35 y, being age >55 y (OR = 0.42, 95% CI: 0.33–0.55, *P* < 0.001) or age 35–55 y (OR = 0.58, 95% CI: 0.48–0.70; *P* < 0.001) were protective factors for LNM.

**Figure 1:**
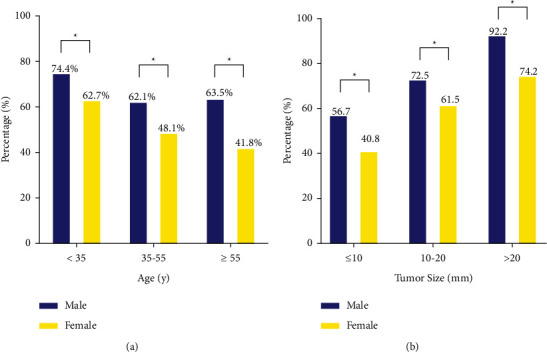
Comparisons of overall lymph node metastasis rates between female and male PTC patients when stratified by (a) age and (b) tumor size.

Comparisons of LNM rates between male and female patients stratified according to age and tumor size group are shown in Figure 1. Clearly, the overall rate of LNM in younger male patients was significantly greatest in all groups. Similar differences were also observed in patients aged 35–55 y (62.1% vs. 48.1%; *P* < 0.001) and patients aged >55 y: (63.5% vs. 41.8%; *P* < 0.001). Furthermore, general trends indicated that lymph node positive rates increased with larger tumors, with male subgroups of tumor size >20 mm having the highest LNM rate of 92.2%. The proportion of positive lymph nodes ranged from 56.7% among male patients to 40.8% among female patients in PTMC (*P* < 0.001).

### 3.3. Subgroup Analysis

**Figure 2:**
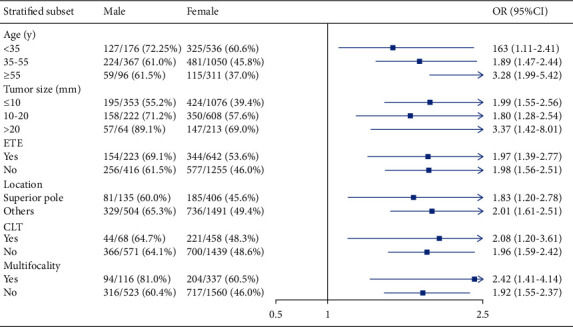
The forest plot for OR comparing central lymph node metastasis between female and male PTC patients according to different variables. ETE, extrathyroid extension; CLT, chronic lymphocytic thyroiditis; OR, odds ratio; CI, confidence interval.

**Figure 3:**
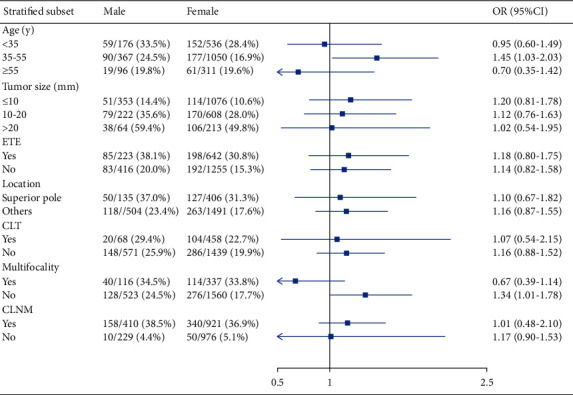
The forest plot for OR comparing lateral lymph node metastasis between female and male PTC patients according to different variables. ETE, extrathyroid extension; CLT, chronic lymphocytic thyroiditis; CLNM, central lymph node metastasis; OR, odds ratio; CI, confidence interval.

We further performed stratified analysis to investigate the associations between gender and CLNM and LLNM among different clinicopathological subgroups. After full adjustment, the stratified analyses indicated that male gender was independently associated with CLNM in all subgroups (Figure 2). However, we observed that there was no significant correlation between gender and LLNM across most subgroups, except for subgroups of patients aged 35–55 y (OR = 1.45, 95% CI: 1.03–2.03) and patients with a solitary tumor (OR = 1.34, 95% CI: 1.01–1.78; Figure 3).

### 3.4. Kaplan–Meier Analyses of Recurrence-Free Survival of Patients

**Figure 4:**
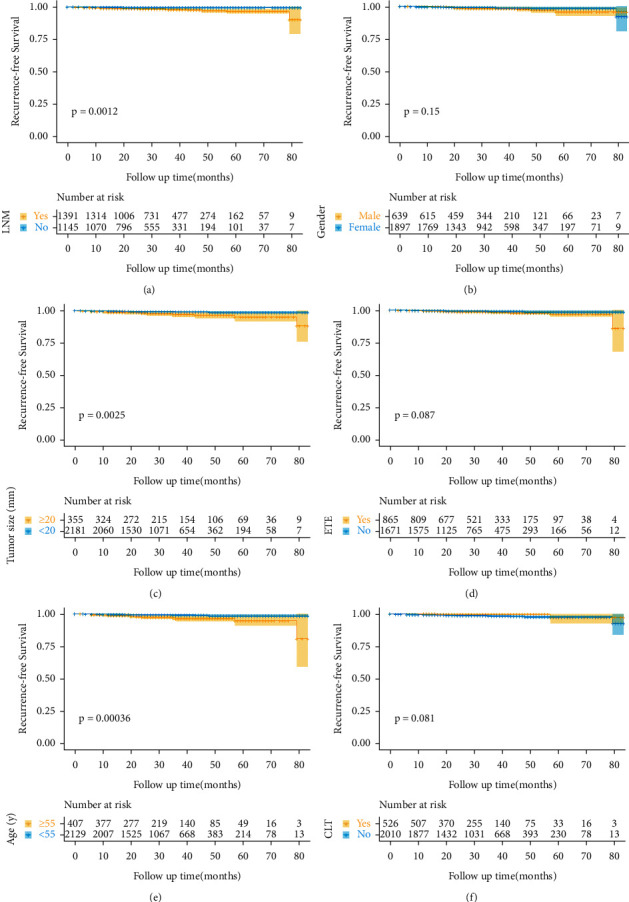
The Kaplan–Meier curves of recurrence-free survival according to (a) lymph node status, (b) gender, (c) tumor size, (d) extrathyroid extension, (e) age, and (f) chronic lymphocytic thyroiditis. LNM, lymph node metastasis. ETE, extrathyroid extension; CLT, chronic lymphocytic thyroiditis.

The median follow-up time was 30 months (interquartile range (IQR) = 18–43). As shown in Figure 4, LNM, age, and tumor size were significantly associated with poorer RFS. However, no differences were found between RFS and genders, ETE, and CLT.

## 4. Discussion

This population-based study in southwestern China elucidated that the prognosis of thyroid cancer is good, and very few patients developed recurrent disease. Males had a higher incidence of LNM, CLNM, and LLNM in PTC than females. Male gender was an independent risk factor of CLNM. After adjustment for confounding factors, the association persisted in all subgroups; whereas, no significant difference of LLNM between female and male patients was found. Additionally, we found male gender was potentially not associated with a worse prognosis.

In this study, univariate analysis showed that there were no differences in terms of age, tumor size, tumor location, and ETE between female patients and males. Nevertheless, the female group had higher percentages of coexistent CLT compared to the male group. This result is consistent with other studies [25, 26].

Surgery is one of the most important treatments for PTC. Poor prognosis can be owing to an inadequate extent of lymph node dissection. Thus, the status of lymph nodes is a crucial factor for guiding treatment options, which may direct the surgeon to adjust the actual extent of surgery [26, 27]. Whether male gender has an influence on LNM of patients with PTC is worthy of attention as, if so, a more aggressive treatment should be adopted. However, aggressive treatment may result in more complications, such as injury of recurrent laryngeal nerves and hypoparathyroidism [9]. It is important to clarify any correlation of gender and LNM. In a research study including 2930 patients with PTC, Lee et al. reported that male gender is a strong independent prognostic factor in patients with PTC, but not in PTMC patients [9]. Nevertheless, our observational study showed that male gender was a strong independent risk factor of LNM not only for PTC but also for PTMC. We further divided the patients into three groups based on tumor size and age to reduce the effect of those two clinicopathological parameters on the correlation between LNM and gender. We observed that the overall rate of lymph node metastasis rose with increasing tumor size, while older age was a protective factor for LNM. Furthermore, the LNM rate in male patients was always higher than in female patients (all *P* < 0.001). In summary, our findings demonstrated that LNM occurred more frequently in young female or male patients with larger tumors. Furthermore, gender may have implications for the surgical management of PTC.

Many studies address the rate and risk factors of CLNM [17, 27, 28]. Sporadic studies indicated an association of male gender with CLNM [24]; however, little attention specifically focuses on this relationship. We conducted a stratified analysis and found that male gender was a stable risk factor for CLNM in all subgroups. In view of this result, we suggest that central lymph node dissection for male patients should be thoroughly and carefully evaluated by the surgeon during surgery. Male gender is considered positively associated with LLNM in some studies [11, 29], while such a relationship is not observed in many other reports [30, 31]. We also investigated the correlation between gender and LLNM. Univariate analysis found that male patients had a higher incidence of LLNM. However, no association was found between gender and LLNM in subgroup analysis after adjustment for other clinicopathological factors. Therefore, a gender factor should not be used to determine the strategy of lateral lymph node dissection.

Many research studies report that LNM is an independent prognostic factor for PTC [21, 32, 33]. Consistent with those studies, we confirmed that LNM was associated with poor prognosis. Male gender is generally thought to be closely related to recurrence of PTC [9, 11, 33, 34]. Ito et al. reported that male gender is a risk factor for lymph node recurrence [35]. Meanwhile, a meta-analysis, including 13 articles, reports that male gender is a poor independent prognostic factor in PTC patients [10]. However, some studies found that male gender is not associated with recurrence-free survival [13, 15, 36, 37]. The Kaplan–Meier analyses of our data indicated that male gender was not correlated with RFS. Based on this, we currently cannot ascertain that gender is or is not an independent survival risk factor in thyroid cancer and that male gender should be taken into consideration as a prognostic factor for risk classification. Certainly, the relatively short follow-up time could have influenced this result; hence, long-term follow-up and Cox proportional hazards regression for adjustment for a large number of confounders is needed to clarify the results. Moreover, future studies are warranted to elucidate the sex-specific molecular mechanisms regarding the role of gender played in tumorigenesis and development of PTC which may assist clinicians with therapeutic decisions [4, 38].

Therefore, while the association of prognosis with gender is controversial, our data provide important evidence to support this issue. The current study utilizes a large sample size to investigate the influence of gender on LNM and risk factors affecting the locoregional recurrence of PTC patients from southwestern China. Furthermore, we have adequately adjusted for confounding factors in multivariable analysis assessing the association between gender and LNM to generate robust results.

The limitations of the study are as follows. First, this was a retrospective study rather than a prospective cohort, so the quality of evidence was limited. Second, due to the short follow-up duration and a considerable portion of early stage PTC, the recurrence outcome was rare. Because of this, we could not perform Cox proportional hazards regression to assess the independent effects of gender on prognosis. A reasonable follow-up is needed to evaluate recurrence. Additionally, this was an observational study of a single center, specifically for a population in southwestern China, which should not be extrapolated to other regions. Hence, it is necessary to use real-world, multicenter data covering a larger number of patients to verify our findings.

## 5. Conclusions

In this observational cohort study of patients with PTC from southwestern China, male gender was independently associated with CLNM but potentially not with LLNM after sufficient adjustment for confounders. Moreover, currently, there is no sufficient evidence to conclude that gender was associated with recurrence. Further long-term follow-up data in regards to recurrence/death are required to assist with risk stratification.

## Figures and Tables

**Table 1:** Demographic and clinicopathologic characteristics of male and female papillary thyroid carcinoma patients.

Characteristics	Total (*N* = 2536), no. (%)	Male (*N* = 639)	Female (*N* = 1897)	*P* value
Age (y)	42.9 ± 11.9	42.7 ± 11.4	42.9 ± 12.2	0.586
<35	712 (28.1)	176 (27.5)	536 (28.3)	0.607
35–55	1417 (55.9)	367 (57.5)	1050 (55.3)	
≥55	407 (16.0)	96 (15.0)	311 (16.4)	
Tumor size (mm)	12.1 ± 8.3	11.9 ± 7.9	12.2 ± 8.4	0.612
≤10	1429 (56.3)	353 (55.2)	1076 (56.7)	0.397
10–20	830 (32.7)	222 (34.7)	608 (32.1)	
>20	277 (11.0)	64 (10.1)	213 (11.2)	
Bilateral				0.614
Yes	393 (13.7)	103 (16.1)	290 (15.3)	
No	2143 (86.3)	536 (83.9)	1607 (84.7)	
Tumor location				0.911
Superior pole	541 (21.3)	135 (21.1)	406 (21.4)	
Others	1995 (78.7)	504 (78.9)	1491 (78.6)	
Multifocality				0.812
Presence	453 (17.9)	116 (18.2)	337 (17.8)	
Absence	2083 (82.1)	523 (81.8)	1560 (82.2)	
Extrathyroid extension				0.630
Presence	865 (34.1)	223 (34.9)	642 (33.8)	
Absence	1671 (65.9)	416 (65.1)	1255 (66.2)	
CLT				<0.001
Yes	526 (20.7)	68 (10.6)	458 (24.1)	
No	2010 (79.3)	571 (89.4)	1439 (75.9)	
LNM				<0.001
Yes	1391 (54.9)	420 (65.7)	971 (51.2)	
No	1145 (45.1)	219 (34.3)	926 (48.8)	
CLNM				<0.001
Yes	1341 (52.5)	410 (64.2)	921 (48.6)	
No	1205 (47.5)	229 (35.8)	976 (51.4)	
LLNM				0.003
Yes	558 (22.0)	168 (26.3)	390 (20.6)	
No	1978 (78.0)	471 (73.7)	1507 (79.4)	
Recurrence	29 (1.1)	11 (1.7)	18 (0.9)	0.131

Continuous data are shown as mean ± standard deviation. CLT, chronic lymphocytic thyroiditis; LNM, lymph node metastasis.

**Table 2:** Univariate and multivariate logistic regression analyses of factors associated with lymph node metastasis.

Variables	Univariate analysis		Multivariate analysis	
	OR (95% CI)	*P*	OR (95% CI)	*P*
Gender (male/female)	1.83 (1.52–2.20)	<0.001	1.93 (1.59–2.35)	<0.001
Age (y)		<0.001		<0.001
<35	Reference		Reference	
35–55	0.56 (0.47–0.68)	<0.001	0.58 (0.48–0.70)	<0.001
≥55	0.46 (0.36–0.59)	<0.001	0.42 (0.33–0.55)	<0.001
Tumor size (mm)		<0.001		<0.001
≤10	Reference		Reference	
10–20	2.24 (1.88–2.67)	<0.001	2.09 (1.75–2.52)	<0.001
>20	4.47 (3.30–6.06)	<0.001	4.18 (3.06–5.73)	<0.001
ETE (+/−)	1.49 (1.27–1.77)	<0.001	1.31 (1.10–1.57)	0.003
Multifocality (+/−)	2.01 (1.62–2.49)	<0.001	1.92 (1.53–2.41)	<0.001
Tumor location				
Superior pole	Reference	0.980		
Others	0.99 (0.82–1.21)			
Bilateral (+/−)	2.24 (1.77–2.83)	<0.001		
CLT (+/−)	0.91 (0.75–1.11)	0.349		

CLT, chronic lymphocytic thyroiditis; ETE, extrathyroid extension; −, negative; +, positive.

## Data Availability

The datasets used and analyzed in the present study are available from the corresponding author upon request.

## Abbreviations

PTC: Papillary thyroid carcinoma
PTMC: Papillary thyroid microcarcinoma
LNM: Lymph node metastasis
CLNM: Central lymph node metastasis
LLNM: Lateral lymph node metastasis
ETE: Extrathyroid extension
CLT: Chronic lymphocytic thyroiditis
RFS: Recurrence-free survival
OR: Odds ratio
CI: Confidence interval.
